# The PTEN Tumor Suppressor Gene in Soft Tissue Sarcoma

**DOI:** 10.3390/cancers11081169

**Published:** 2019-08-14

**Authors:** Sioletic Stefano, Scambia Giovanni

**Affiliations:** 1 UOC Anatomia Patologica, San Camillo De Lellis, 02100 Rieti, Italy; 2 UOC di Ginecologia Oncologica, Dipartimento di Scienze della Salute della Donna e del Bambino e di Sanità Pubblica, Fondazione Policlinico Agostino Gemelli IRCCS, Largo A. Gemelli 8, 00168 Rome, Italy; 3 Istituto di Clinica Ostetrica e Ginecologica, Università Cattolica del Sacro Cuore, Largo F. Vito 1, 00168 Rome, Italy

**Keywords:** PTEN, Soft tissue sarcoma, liposarcoma, leiomyosarcoma, malignant peripheral nerve sheath tumor, undifferentiated pleomorphic sarcoma, myxofibrosarcoma, gastrointestinal stromal tumor, epithelioid sarcoma, synovial sarcoma

## Abstract

Soft tissue sarcoma (STS) is a rare malignancy of mesenchymal origin classified into more than 50 different subtypes with distinct clinical and pathologic features. Despite the poor prognosis in the majority of patients, only modest improvements in treatment strategies have been achieved, largely due to the rarity and heterogeneity of these tumors. Therefore, the discovery of new prognostic and predictive biomarkers, together with new therapeutic targets, is of enormous interest. Phosphatase and tensin homolog (PTEN) is a well-known tumor suppressor that commonly loses its function via mutation, deletion, transcriptional silencing, or protein instability, and is frequently downregulated in distinct sarcoma subtypes. The loss of PTEN function has consequent alterations in important pathways implicated in cell proliferation, survival, migration, and genomic stability. PTEN can also interact with other tumor suppressors and oncogenic signaling pathways that have important implications for the pathogenesis in certain STSs. The aim of the present review is to summarize the biological significance of PTEN in STS and its potential role in the development of new therapeutic strategies.

## 1. Introduction

Soft tissue sarcomas (STSs) constitute a heterogeneous group of rare solid tumors of mesenchymal tissue origin, with more than 50 different histological subtypes and distinct clinical and pathological features [1]. Collectively, these tumors account for approximately 1% of all adult malignancies and 15% of pediatric malignancies. The therapeutic options are determined based on the histology (subtype, grade), localization, resectability, and presence or absence of metastases.

The mainstay treatment of the primary STS is wide local excision with or without complementary radiotherapy (RT) to improve local control and eventually chemotherapy [2]. In specific groups of sarcomas (high grade, >5 cm, and deep-seated), adjuvant chemotherapy with anthracyclines and ifosfamide is a standard choice of treatment [3]. The most common sites of origin are the extremities; however, STSs can arise in any part of the body, including the abdomen and head and neck region [4]. They most commonly metastasize to the lungs and less frequently to the liver peritoneum and bones.

Their relatively low incidence, compared to other solid tumors, and their heterogeneity, make it difficult to study and evaluate a high number of specific subtypes of STS. These difficulties limit the ability to conduct traditional clinical trials, establish advanced drug development, and determine rationale treatment strategies in the advanced stage.

The current therapies allow a local control in about 80–90% of patients; however, approximately half of the patients with high-grade tumors develop metastatic disease [5], with an estimated 3-year survival range from 20% to 45% [6].

**Figure 1:**
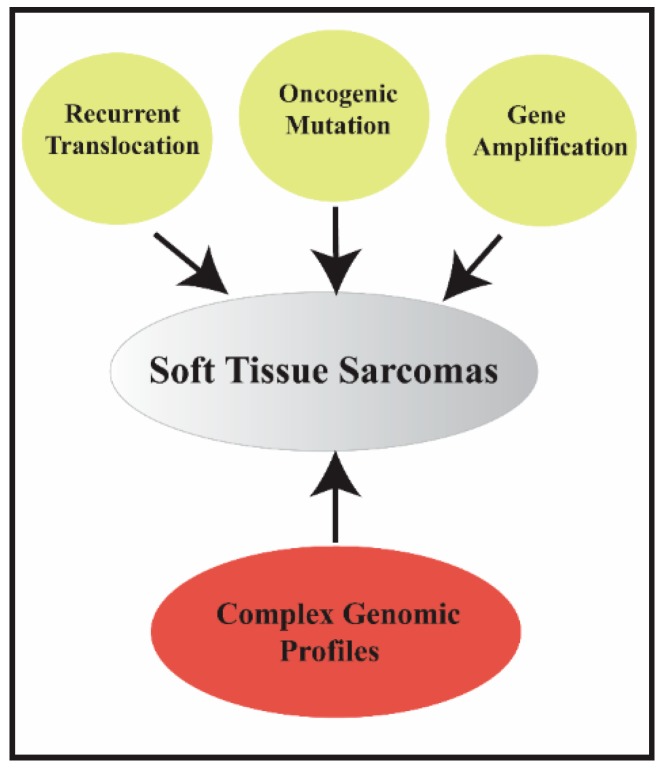
Types of molecular alterations in soft tissue sarcomas.

The systematic therapy in locally advanced and metastatic STS is based on standard chemotherapy with anthracycline (e.g., doxorubicin). Although there is no formal demonstration that multi-agent chemotherapy is more effective than single-agent chemotherapy in terms of overall survival (OS), anthracyclines plus ifosfamide is considered, in most cases, the treatment of choice [7]. Due to the limited number of cases, most of the clinical trials, up until the last decade, were organized without considering the underlying distinctive molecular events, with some notable exceptions, such as imatinib in gastrointestinal stromal tumors [8]. Recently, several clinical trials tried to address this issue, focusing on a more histology- and molecular-based clinical approach [9,10,11]. A recent multicenter clinical trial used a histology-driven neoadjuvant chemotherapy regimen, concluding that further studies are necessary to analyze the highly specific group of STSs [10]. Sarcomas are widely believed to develop as a result of genetic alterations in mesenchymal progenitor/stem cells, but the precise cellular origin of most of these tumors remains unknown. Based on molecular/cytogenetic evaluation, these tumors can be divided into two main categories: sarcomas with specific and distinct genetic alterations and sarcomas displaying multiple and unspecific complex karyotypic abnormalities (Figure 1).

**Figure 2:**
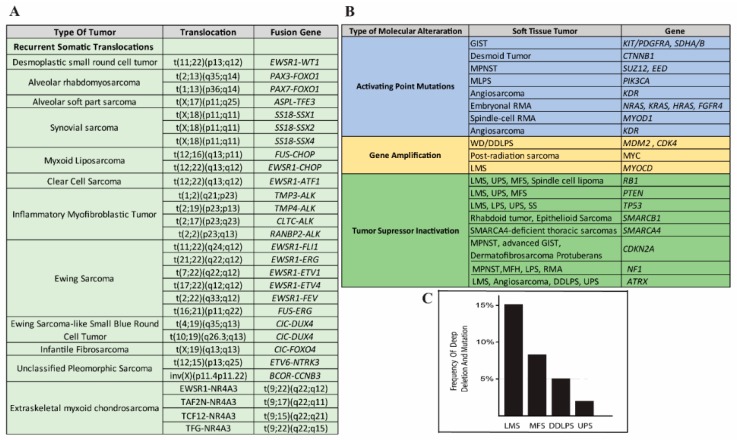
(**A**) Adapted from [12] common translocations associated with subgroups of soft tissue sarcomas. (**B**) Adapted from [13] distinctive molecular abnormalities in major soft tissue sarcoma subtypes. (**C**) Phosphatase and tensin homolog (PTEN) gene alterations (mutations and deep deletions) rate of the most frequent sarcoma subtypes according to the Cancer Genome Atlas (TCGA) study [14] (adapted from www.cbioportal.org).

**Figure 3:**
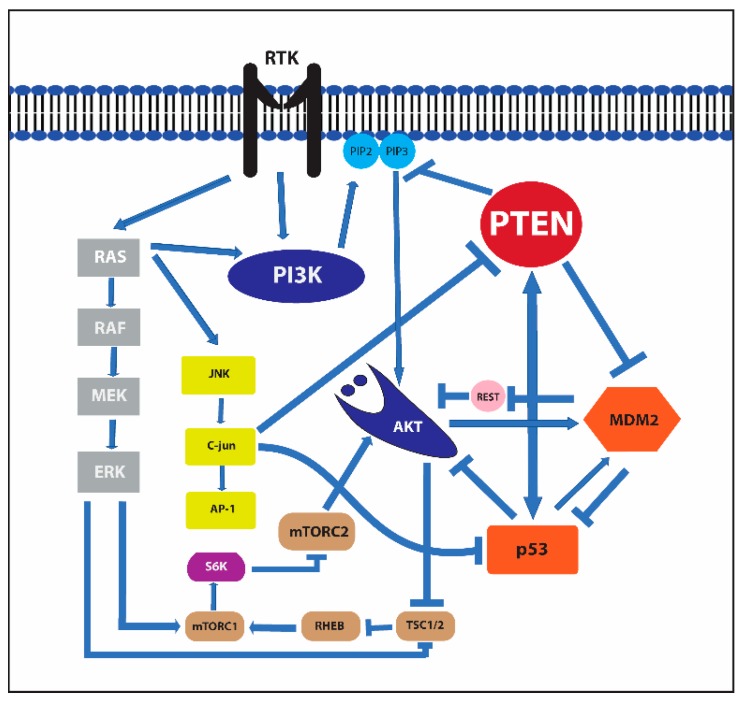
Receptor tyrosine kinase, murine double minute 2 (MDM2), mammalian target of rapamycin (mTOR), and c-Jun N-terminal kinase (JNK) signaling pathways and their interactions.

The first group of tumors can be subcategorized into three further subgroups: (1) sarcomas, accounting for 15–20% of cases, carrying a recurrent translocation such as FUS-CHOP in myxoid liposarcoma (MLPS) and SS18-SSX in synovial sarcoma (SS) (Figure 2A); (2) sarcomas with mutations on specific genes, such as c-KIT or platelet-derived growth factor receptors (PDGFR) mutation in gastrointestinal stromal tumor (GIST) (Figure 2B); (3) sarcomas with distinct amplification, such as the 12q14-15 amplicon in well-differentiated liposarcoma (WDLPS). Dedifferentiated liposarcoma (LPS) (DDLPS) is a high-grade sarcoma with complex genomic alterations, but shares the same amplicon of WDLPS. The second group of sarcomas accounts for 50–60% of all tumors and harbors a highly complex profile represented by several gene losses and gains of numerous chromosomes or chromosome regions and amplifications. These tumors are mainly represented by leiomyosarcoma (LMS), myxofibrosarcoma (MFS), malignant peripheral nerve sheath tumor (MPNST), and unclassified pleomorphic sarcoma (UPS).

The refined understanding of the mechanisms that drive tumorigenesis in some STSs, in addition to enrichment of the diagnostic classification, has made the identification of potential targets possible for an improved treatment approach. Translational research has also aided in improving the knowledge of molecular patterns and helped to explore key interactions between significant pathways.

Several sarcomas carry abnormalities in well-known tumor suppressor genes, such as retinoblastoma (RB), p53, phosphatase, and tensin homolog (PTEN) [14,15], and other members of growth-factor signaling pathways [16]. The most common growth-factor pathways that seem to be activated in specific types of sarcomas include the insulin-like growth factor 1 (IGF1)-receptor [17,18], the PDGFR [15,19], the c-KIT receptor [20,21], and the c-MET-receptor [22,23]. Activating mutations in growth factor receptors leads to activation of the phosphatidylinositol 3′ kinase (PI3K)/Akt/mammalian target of the rapamycin (mTOR) pathway [16].

PTEN is a well-known tumor suppressor, located in chromosome 10q23, and encodes a 403 amino acid protein that has both phosphatase-dependent and -independent functions.

It has an essential role in the PIK3/PTEN/Akt/mTOR pathway through the main catalytic function of the dephosphorylation of phosphatidylinositol 3,4,5 trisphosphate (PIP3), which is a strong activator of 3 phosphoinositide-dependent kinase (PDK) and Akt. Activation of PI3K by receptor tyrosine kinases elicits the activation of mTOR complex 1 and 2 (mTORC1 and mTORC2) and the promotion of cell growth, proliferation, motility, and survival [24,25] (Figure 3).

This pathway, due to its role in a wide range of key cellular processes, has been intensely studied for its involvement in tumor initiation and progression and is frequently dysregulated in cancer through the increased expression and/or activation of receptor tyrosine kinases, mutations in pathway components, or the loss of negative pathway regulators. Therefore, the role PTEN, one of the central regulators of this signaling cascade, is crucial and the loss of its function leads to increased levels of PIP3 and upregulates the PI3K–Akt pathway that stimulates cell growth and survival [26].

The first studies that confirmed the 10q deletion/mutation and the consequent PTEN downregulation were performed in brain, bladder, and prostate cancer and, subsequently, a partial or complete loss of this gene has been discovered in several more cancer subtypes. Although PTEN functional loss is known to occur at a significant rate in the majority of human tumor subtypes [27], inactivating mutations of PTEN only occur in a fraction of PTEN-deficient tumors.

Loss of PTEN function can result from several non-genomic mechanisms, such as epigenetic and transcriptional silencing, post-translational modifications (phosphorylation, acetylation, oxidation, and ubiquitylation), or by interaction with proteins that suppress its functions. Although there is a vast spectrum of research regarding PTEN and its role within epithelial and hematological tumors, sarcoma studies in this field are not so extensive (Table 1). A recent review profiled the data of the protein expression, amplification/translocation, and DNA sequencing of 2539 bone and STSs and concluded that the loss of PTEN expression is present in 38.6% of tumors, most commonly in leiomyosarcomas (LMSs), epithelioid sarcomas, alveolar rhabdomyosarcomas, osteosarcomas, and chordomas [15]. The mutations and deletions of the PTEN gene occur in 2% to 10% of STSs [28], with an overall mutation/deletion rate in the PI3Kinase pathway of 20% [29].

Despite the relatively low incidence of the PTEN mutation rate, there is an increased activity in several of its downstream factors and multiple studies suggest a possible role for Akt activation in several STSs [14,30,31,32,33]. For example, an increased expression of Akt has been observed in LMS, DDLPS, and UPS [30]. Additionally, in a large panel of STSs, phosphorylated Akt (pAKT) expression by immunohistochemistry (IHC) has been shown to have some prognostic significance for both disease-free and overall survival [31].

In the struggle to understand the complex mechanism of sarcomagenesis and the development of new therapeutic strategies, the role of PTEN and its interaction with other altered pathways in distinct subtypes of STSs could be crucial. Therefore, we separately discuss the oncogenic pathways of the most common STSs and their interaction with the PIK3/PTEN/AKT/mTOR pathway.

## 2. Leiomyosarcoma

Leiomyosarcoma (LMS) is a type of STS with distinct features of smooth muscle differentiation and accounts for 15–20% of all STSs. It commonly involves the retroperitoneum, a deep extremity, the uterus, blood vessels, and superficial dermis. Uterine leiomyosarcoma (uLMS) is the most frequent LMS [47]. The overall prognosis is poor, with local recurrence and/or metastasis in approximately 40% of cases, despite optimal loco-regional treatment.

LMSs are defined by large genomic imbalances and aberrant karyotypes with several molecular alterations [34]. High-grade tumors contain significantly more DNA copy number gains, while low-grade tumors include more copy number losses [48], with a p53 mutation range of 24% to 39% [49] and murine double minute 2 (MDM2) amplification rate of 14% [50]. However, no recurrent molecular target/biomarker useful in the routine prognostication or determination of treatments has been discovered.

Cytogenetic studies have shown that almost half of the LMSs (40–50%) harbor 13q and 10q loss that contains retinoblastoma 1 (RB1) and PTEN genes, respectively [34,51,52]. The recent Cancer Genome Atlas (TCGA) study, which analyzed the deletion rate according to the Genomic Identification of Significant Targets in Cancer (GISTIC), reported that 13% of LMS samples had a deep deletion (possibly homozygous) and 68% had a shallow deletion (possibly heterozygous) of the PTEN gene, while PTEN mutations were detected in 5% of tumor samples (Figure 2C) [14]. High-density microarrays showed that many upstream modulators or intrinsic components of the PI3K-Akt pathway are over-expressed [53] and in most cases, a correlation exists between PTEN alteration and high signaling of the PI3K/mTOR pathway [14].

The PTEN locus, even when it is lost or mutated, does not correlate with the protein expression either by IHC or by western blot analysis. The discrepancy of comparative genomics and western blot analysis, in these studies, was as much as 46% of cases [35]. The authors suggested that the reason for this in some of these tumors could be technical, like the cross-reaction of the antibody in IHC with a smooth muscle antigen or the presence of numerous vessels and endothelial cells that retain PTEN expression and interfere with the quantification of PTEN loss in western blot analysis and/or comparative genomic hybridization [35]. However, in most cases, the reason was unidentifiable, confirming the difficulty in assessing the PTEN gene expression in the context of tumors with a complex genome.

Hernando et al. reported, in their mice model with conditional smooth muscle-cell-specific PTEN knockout, widespread smooth muscle hyperplasia and hyperactivation of Akt signaling, which caused malignant progression to LMS in 80% of cases through the release and activation of mTOR via TSC2 [30]. They observed that the absence of PTEN is necessary for tumor development, but it is not sufficient for the progression to malignancy and additional steps like p53 suppression are required. Furthermore, MDM2 was overexpressed in LMS compared to the hyperplastic smooth muscle, which can result from the Akt activation that induces MDM2 expression and the subsequent p53 inactivation. These mice were treated with an mTOR inhibitor (rapamycin) that led to a significant decrease of pAkt and pS6, followed by an increase in the life span and a decrease in tumor growth, highlighting the crucial role of this pathway in LMS tumorigenesis. Therefore, PTEN loss in LMS is an early event that precedes oncogene activation by paving the way for genomic instability and malignant progression, presumably by a “gene-dose effect” [52].

Gibault et al. observed that in some high-grade LMS with a low level of PTEN, the expression of Akt and mTOR is not increased as expected. However, in well-differentiated LMS, Akt activation is persistently observed in PTEN-deleted tumors [35,43]. Another study that analyzed a large cohort of uterine LMSs detected an increased phosphorylation of S6 in serine 240/244 (p-S6^S240^) in 29% of the samples. Moreover, the same study showed an increased phosphorylation of S6^S240^ in high-grade LMS samples compared to the low-grade LMSs [36].

It was also observed that rapamycin-insensitive companion of mammalian target of rapamycin (RICTOR) the major component of the mTOR complex 2, is overexpressed in well-differentiated LMSs [35]. This protein has an essential role in smooth muscle differentiation through its function in actin polymerization and cytoskeleton organization [54]. Moreover, through its kinase activity of Akt1 phosphorylation on serine 473, it can be part of the oncogenic process.

Studies have confirmed that estrogen (ER) and progesterone (PgR) expression is positive in 25–60% and 35–60% of uLMS samples, respectively, compared to non uLMS samples, which showed a positive expression for both ER and PGR in a range of 15% to 25%, with a gender-defining prevalence [55,56].

Estrogen plays an important role in tumor cell proliferation through two estrogen receptors, estrogen receptor (ER) α and ER β, which are ligand-dependent transcription factors that belong to the superfamily of nuclear receptors.

Research on more common hormone-dependent solid tumors, such as endometrial and breast cancer, has indicated cross-talk between the Akt/PTEN pathway and ERα [57,58,59]. In vitro studies on endometrial cancer showed 17β-estradiol (E2), signaling a decrease in PTEN phosphorylation and its stability and activity through ERα signaling, so a rapid increase of E2 decreases PTEN activity [60]. In breast tumors, ERα binds to the p85 regulatory subunit of PI3K in a ligand-dependent manner, thereby activating Akt and subsequently its downstream effectors [61]. All these findings indicate that the PI3K-involved signaling system may be related to the biology of ERα and E2 exposure and may increase the risk of hormone-dependent cancers.

Despite all the evidence of PTEN/Akt/mTOR pathway alterations, initial clinical results with the first-generation mTOR inhibitors were not encouraging. The reason for this can be found, as in other types of cancers, in their exclusive function of inhibition of mTORC1, which may lead to the feedback activation of Akt and sustained tumor growth signaling through mTORC2 [62]. Additionally, it triggers the negative feedback control mechanism of the MAPK pathway [63,64,65], which hyperactivates the RTK/IRS-1/PI3K pathway and increases the signal toward the RAS-Raf1-MEK1/2-ERK pathway.

New-generation compounds can inhibit PI3K and mTOR and consequently avoid Akt activation through the mTORC1- or mTORC2-mediated negative feedback loops [63]. In vitro studies with dual inhibition of PI3K and mTOR have established a strong anti-tumor activity in LMS, which was seen to be significantly higher than either agent alone. However, these combinations showed only limited benefits in time, due to the relatively rapid acquisition of drug resistance; indeed, although there is a thorough inhibition of mTORC1, mTORC2, and PI3K, an over-activation of the RAS/MEK/ERK pathway has been noted. This increase can be explained by the observation that dual inhibition of the PI3K/mTOR in LMS enhances the ERK pathway through the inhibition of a PI3K-independent feedback loop involving mTORC2 [65].

## 3. Liposarcoma

Liposarcoma (LPS) is the most common STS, accounting for up to 25% of all STS in adults [1]. It is thought to originate from mesenchymal stem cells within the adipose tissue [66,67,68] and it is classified into three distinct entities: well-differentiated/dedifferentiated (WDLPS/DDLPS), pleomorphic (PLPS), and myxoid (MLPS) [1] LPS.

WDLPS represents more than 40% of all the diagnosed LPS. Although this tumor does not metastasize, it poses a significant risk of local recurrence and up to a 20% possibility of de-differentiating [69,70]. DDLPS is a high-grade sarcoma morphologically resembling a non-lipogenic undifferentiated pleomorphic sarcoma juxtaposed to WDLPS. About 90% of DDLPS arises de novo, while 10% occurs in recurrences of WDLPS. It has a more aggressive behavior than WDLPS, with an estimated 5-year disease-specific-survival of 44% versus 93% [71].

Both WDLPS and DDLPS are characterized by the presence of supernumerary ring and/or giant rod chromosomes containing amplified segments from the 12q13–15 region [72]. Several oncogenes reside in this region, including MDM2, CDK4, and HMGA2, with a critical role in the cell cycle.

MDM2 (murine double minute 2), is always amplified and overexpressed in these tumors and has a well-established role in the pathogenesis of these STSs. The encoded protein is an established negative regulator of the tumor suppressor p53, a transcription factor and a major tumor suppressor, that controls G1 and G2/M cell cycle checkpoints and impacts the biological process related to cell development, growth control, and apoptosis [73].

MDM2 and P53 interact through an autoregulatory negative feedback loop as P53 stimulates the expression of MDM2 and, in turn, MDM2 inhibits p53 activity by stimulating its degradation in the nucleus and cytoplasm, blocks its transcriptional activity, and promotes its nuclear export. As a result, p53 is kept at low levels in the absence of stress signaling [74]. When DNA damage occurs in a normal cell, either by exogenous or endogenous factors, p53 is phosphorylated on several amino acid residues, and consequently, MDM2 can no longer interact with p53, allowing p53 to perform its tumor-suppressive functions [75]. In LPS, MDM2 amplification and the consequent protein overexpression inactivate the function of p53, allowing cells to escape from the usual growth constraints and permitting the tumor to harbor more genomic alterations from one cell generation to the next.

P53 and MDM2 are linked to PTEN and the PI3K/Akt/mTOR pathway, playing a prominent role in the regulation of pro-apoptotic and anti-apoptotic signals (Figure 3).

Akt can phosphorylate serine 166 and serine 186 in the domain of MDM2 that contains a nuclear localization motif [76,77]. The phosphorylation of these amino acids allows the protein to move from the cytoplasm to the nuclei and interact with p53. Furthermore, AKT-mediated phosphorylation of MDMX at Ser367 also stabilizes MDM2 E3-ligase activity [78]. Additionally, MDM2 prevents the localization of the repressor element-1 silencing transcription factor (REST), a tumor suppressor that downregulates PI3-kinase activity and Akt phosphorylation on the p85 promoter. Therefore, it induces an opposing function with the reduction of p85 expression and the increase of Akt phosphorylation [79].

Despite the upregulation of the Akt pathway in several LPS samples [30,37], the Cancer Genome Atlas (TCGA) study showed PTEN mutation/deletion in 5% of the DDLPS samples that were analyzed [14] (Figure 2C). Utilizing a novel DDLPS xenograft mouse model to analyze gene expression profiles from patient-derived tumors, Smith et al. [80] observed that the PTEN locus in primary human tumors did not show any genetic alterations; however, the expression analysis showed a significant decrease of the PTEN level compared with lipoma samples. Additionally, they showed that PTEN expression in patient samples correlates with poor disease-specific survival (DSS) [80]. Together, these results suggest that the PTEN-controlled PI3K/Akt/mTOR pathway is a potential prognostic signature and therapeutic target for DDLPS.

PTEN regulates the function of p53 through the dephosphorylation of PIP3, which blocks the nuclear entry of MDM2 and causes its degradation by restricting it to the cytoplasm. Therefore, PTEN protects p53 from MDM2-mediated degradation [81].

The PTEN promoter also contains a p53 functional binding site by which p53 induces transcription of the PTEN gene and elevates cellular levels of the PTEN protein [82]. Therefore, it protects itself from overly robust survival signals.

The inhibition of PI3K/ Akt/mTOR signaling can augment P53-mediated apoptosis, induced by the MDM2 antagonist, in LPS cell lines. These results are in agreement with studies on several epithelial tumors [83] which indicate that inhibition of the PI3K pathway, including PI3K itself, Akt, or mTOR, can enhance the activity of MDM2 inhibition.

To elucidate the importance of the Akt pathway in WDLPS, Gutierrez et al. [37] established a zebrafish model with constitutively active Akt2 in mesenchymal progenitors that developed a tumor histologically resembling a WDLPS. P53 homozygous mutant models showed a significant incidence of developing a tumor (29%) compared to p53 wild-type (8%) and p53 heterozygotes (6%), theorizing that both Akt pathway activation and p53 inactivation play an important role in the development of LPS.

Multiple in vitro and in vivo studies have investigated the effects of rapamycin, and a therapeutic resistance through a feedback-dependent activation of upstream Akt has been observed. These results suggest both the mTORC1-dependent and -independent role of the PI3K-Akt pathway in the pathobiology of WDLPS and DDLPS [37,84,85,86].

BEZ235, a dual PI3K/mTOR inhibitor [87], was more effective and silenced the Akt pathway activation in LPS cell lines and induced G1 arrest and apoptosis compared to rapamycin [37]. As a result, the multi-kinase, dual-action inhibitor, sorafenib, was investigated in phase 2 trials for advanced STS, including LPS; however, a lack of significant clinical efficacy was observed [88].

C-JUN is a signal-transducing transcription factor and an important member of the AP-1 family that is involved in cell cycle regulation. It is amplified and over-expressed in ~20% of DDLPS [66,68] and is associated with a high malignant potential [14,89].

The overexpression of C-JUN in LPS enhances Akt activity as C-JUN suppresses PTEN transcription by binding to PF1, a site of activator 1 (AP-1) [90]. Additionally, one of the essential downstream signaling pathways of Akt is c-Jun N-terminal kinase (JNK), which belongs to the family of mitogen-activated protein kinase (MAPK) [91]. The interaction between these two pathways may determine the fate of the cell between survival or apoptosis since JNK also has an important role in apoptosis.

MLPS is the second most common LPS after WDLPS, comprising around 30–35% of all LPS. Morphologically, these tumors include a broad spectrum of lesions from a less cellular myxoid variant to a more aggressive, highly cellular, round cell LPS. Over 90% of these sarcomas have a pathognomonic *t*(12;16) (q13;p11) translocation that results in expression of the FUS-DDIT3 fusion protein, whereas they less commonly carry *t*(12;22), resulting in the expression of the EWSR1-DDIT3 fusion protein. Gene expression studies in MLPS have identified the recurrent upregulation of *MET*, *RET*, and *PIK3CA.* Therefore, it is conceivable that these oncogenes are under the downstream transcriptional control of the fusion proteins [53]. It has been observed that the frequent p110α catalytic subunit mutations of PI3K are associated with a poor prognosis. Additionally, the amplifications and the mutations, mostly in exon 20 and exon 9, of PIK3Ca and PTEN loss, have been reported in 14–18% and 12% of cases, respectively [53].

Tumors with a round cell morphology are more likely to have a strong membranous IGFR1 expression or an activation mutation of PIK3CA compared to the less cellular myxoid variant, suggesting that these alterations are more common in aggressive subtypes with a poorer outcome. There is a significant increase of p4EBP1 in round-cell LPS compared to the myxoid LPS, which is closely related to activating events, such as PTEN loss, IGF1R expression, or the mutation of PIK3CA [38].

Although molecular results are suggesting a deregulation of the PTEN-Akt-mTOR pathway in these tumors, the limited number of patients that have been treated with the mTOR inhibitors have not shown a significant response. A study with four patients treated with everolimus (three patients) or temsirolimus (one patient) showed only a minor response, with a decreased tumor density in two patients. Interestingly, the molecular analyses of one of these patients identified an inactivating mutation in PTEN [92].

## 4. Malignant Peripheral Nerve Sheath Tumor

The malignant peripheral nerve sheath tumor (MPNST) is a subgroup of STS mostly, but not exclusively, arising in the nerve sheath and accounting for roughly 2% to 5% of sarcomas [93,94]. The cell of origin remains unclear, but it is hypothesized to be of neural crest lineage [95]. MPNSTs are detected in three very distinct clinical contexts, raising the suspicion of three distinct subtypes arising by different pathogenic processes. Approximately 40–47% of all MPNSTs occur sporadically, whereas 40–50% are detected in the setting of neurofibromatosis type 1 (NF1) syndrome. This autosomal dominant disease is caused by mutations of the NF1 gene located at chromosome 17q11.2, which encodes the neurofibromin, a protein involved in the regulation of several cellular signaling pathways and responsible for cell proliferation and differentiation. The patients with NF1 develop multiple neurofibromas and a benign tumor, and carry an estimated 8% to 13% lifetime risk of developing MPNST [96,97]. The genomic profile of the neurofibromas is simpler compared to MPNSTs, which shows multiple chromosomal gains and losses [98,99]. The remaining MPNSTs (10%) arise secondary to previous exposure to radiation therapy.

The most commonly altered molecular pathways in MPNSTs are the p19^ARF^ -MDM2-p53 (p53 mutation in 75%) [100] and p16^INK4a^-cyclin D-Rb (CDKN2A and Rb loss in 50% and 25%, respectively) [101,102].

Loss of neurofibromin is considered a tumor-promoting event that leads to RAS hyperactivity and the consequent activation of multiple downstream survival and proliferative pathways, including MAPK, mTOR, and Akt [99]. Although RAS activation, through biallelic loss of neurofibromin, is considered to be directly responsible for the development of neurofibromas in NF1 syndrome, this is not sufficient to develop the malignant counterpart of MPNST.

The reduction or loss of PTEN has been reported in a subset of MPNSTs, both in human and animal models [39,40,103,104,105]. Bradtmoller et al. observed a significantly reduced median proportion of PTEN positive cells (30% of cells) in human MPNST samples compared to neurofibroma (5% of cells) samples [39]. IHC has also shown that the phosphorylated S6 (pS6, Akt (pAkt) and Erk (pErk) are more expressed in MPNSTs than neurofibromas [40]. In addition, mTOR and its downstream targets, such as p4EBP1 and the Ps6 ribosomal protein, are increased compared to the benign counterpart [106]. PTEN protein expression is correlated with improved disease-specific survival (DSS) and disease-free survival (DFS) [107]. A higher methylation frequency in MPNST suggests that the methylation of CpG island 3’ is one of the mechanisms that down-regulate PTEN in MPNST [39].

A transgenic mouse model has been created, lacking both PTEN and NF1 in Schwann cells and in their precursor, that developed a high-grade peripheral nerve sheath tumor, confirming the importance of PTEN in tumor development and progression from neurofibroma to MPNST [103]. The concomitant activation of the K-RAS oncogene along with the single allelic deletion of PTEN led to a 100% penetrable development of NF1 lesions and subsequent progression to MPNST in mice [40].

Several preclinical studies have analyzed the possible clinical implications of these findings. The MTORC1 inhibitor showed some promising effects in various cell lines [108,109,110]. However, the effects of mTORC1 inhibitors were cytostatic rather than cytotoxic and were transient with tumor re-growth during and/or after discontinuation of the therapy [106,108,110]. The reason for this can be explained with the feedback loop by which mTORC1 inhibition promotes the activation of the pro-tumorigenic PI3K/Akt survival pathway. The use of dual compounds of mTORC1 and PI3K/Akt [106] or mTORC1/2 inhibition [111] had some better effects on the culture. Currently, several mTOR inhibitors and their derivatives, such as everolimus and temsirolimus, are combined with other drugs in preclinical and clinical trials [112,113,114]. Studies are pointing out that RAF inhibitors combined with mTOR/Akt or MEK inhibitors can also be potentially useful, but further clinical trials and studies are necessary to confirm this.

## 5. Undifferentiated Pleomorphic Sarcoma/Myxofibrosarcoma 

Undifferentiated Pleomorphic Sarcoma (UPS) is a rather new term introduced in 2013 WHO classification and comprises a heterogeneous group of tumors. It is the fourth most common subgroup, with an incidence of 15–20% of all STSs. This category recognizes those tumors that are not classifiable in a distinct category by current molecular and immunohistochemical criteria and that do not have any proven line of differentiation [94]. Therefore, it is considered by the pathologists and the clinicians to be a diagnosis of exclusion. Most of these tumors were previously classified as malignant fibrous histiocytoma (MFH), which was subsequently renamed as UPS.

These sarcomas are usually very aggressive and can arise from any part of the body, with a high local recurrence and metastasis. Cytogenetically, UPSs are aneuploid tumors without recurrent or characteristic genetic abnormalities. The absence of a precise histogenesis and a distinct molecular pattern represents a significant diagnostic and therapeutic challenge.

Myxofibrosarcoma (MFS), with the exception of the presence of a myxoid component, shows similar morphological features of UPS. However, due to some distinct clinicopathological features, it has been classified as a separate clinical entity. MFS, compared to the UPS, is often lower grade, with a higher local recurrence rate and variable metastatic risk based on the grade (low grade less prone to metastasis, while high grade similar to UPS). The UPS, compared to MFS, is more cellular, with a higher incidence of distant metastasis and a worse survival rate. The Cancer Genome Atlas (TCGA) study confirmed that from the molecular point of view, MFH and UPS tumors could be considered as a single spectrum of disease [14]. Therefore, we discuss these tumors in the same chapter.

Several studies established that UPSs and LMSs, compared with other sarcoma subtypes, also exhibit similar profiles of recurrent chromosomal imbalances [43,115,116,117], with some genomic imbalances being more frequently detected in UPS [118]. In a recent study based on gene expression profiling by transcriptome analysis on a large cohort of sarcomas with complex genomics, a 10 q loss was found in a subset of STS that included UPS and MFS samples. [43]. Immunohistochemical evaluation of the pAkt in UPS also showed an increased expression of this protein [30]. The high pAkt levels were associated with a poorer disease-specific survival (DSS) outcome, independent of other clinical variables (histological grade, site and depth of the tumor, positive/negative margins, gender and age of patients) and molecular factors (expression of phosphorylated FKHR, phosphorylated ERK1/2, Hepatocyte Growth Factor and C-Met) [30,119,120]. The PTEN gene deletion in UPS is 4% and in MFH is 8% [14] (Figure 2C), suggesting a PTEN-independent activation of the Akt/mTOR pathway [120]. For example, it cannot be excluded that the oncogenic activation of this pathway can occur through an inappropriate or unregulated activation of the upstream RTKs or by cross-talk of other pathways with members of the Akt/mTOR pathway. In vitro studies have indicated that a combined inhibition of PI3K and mTOR has a strong antiproliferative effect at low nanomolar concentrations because of cell-cycle arrest in the G1 phase and the induction of apoptosis; however, an in vivo model compared with the non-treated tumors did not show any change in cell proliferation and apoptosis [121]. The potential role of the deregulation of PI3K/Akt/mTOR in UPS makes this pathway a potential therapeutic target, but each tumor, given their heterogeneity, has to be evaluated as a unique molecular entity.

## 6. Synovial Sarcoma

Synovial sarcoma (SS) is accounting for approximately 8% to 10% of all STSs [122]. Although the name implies an association with the synovium, its origin is unclear. SSs in >95% of cases harbor a balanced t(X;18)(p11.2; q11.2) chromosomal translocation that produces an SS18-SSX fusion oncogene [1]. Additional somatic mutations are relatively scarce [123]. Studies have established that, although SS18-SSX is sufficient to drive synovial sarcomagenesis in mice [124], these models of tumors did not reflect the full biological potential of SS. These findings encouraged the research to discover additional genetic or epigenetic changes necessary for tumor progression and metastasis [125].

It has been suggested that the biology of the SS18:SSX fusion protein can be altered according to associated genomic alterations, such as p53, H-RAS mutation, MDM2 amplification [126], and PTEN deregulation [127]. P53 mutation has only been identified in 16% of these tumors, but in wild-type p53, its function may be impaired through upstream regulatory events, including Akt–PTEN pathway deregulation [44].

PTEN mutation occurs in 7–14% of tumors [44,45] and PIK3CA is mutated in only a few cases [45], but immunohistochemical studies have detected a strong expression of pAkt and mTOR [128,129].

Molecular studies have discovered mutations in catenin beta 1 (CTNNB1) and adenomatous polyposis coli (APC) in 8% and 13% of cases, respectively [44,130,131], providing some support for the oncogenic activation of the Akt-mTOR and WNT signaling pathways.

It has been observed that PTEN deletion promotes metastasis and angiogenesis in mice bearing conditional expression alleles of SS18-SSX1 or SS18-SSX2 [127]. Another study with human tumor samples using IHC showed that PTEN is lost in almost 40% of SSs. Patients whose tumors showed a loss of PTEN expression had a shorter overall survival [45].

It has been noted that the transcription factor EGR1 is maintained at a low level through the SS18-SSX oncoprotein, enabling the tumor to escape the apoptotic mechanism. The modulation of EGR1 through the histone deacetylase (HDAC) inhibitor and its increase induce PTEN expression and consequent cell death [132].

## 7. Gastrointestinal Stromal Tumor

Gastrointestinal stromal tumor (GIST) is the fifth most common STS and the most common mesenchymal sarcoma located in the gastrointestinal tract [20].

The majority (75–80%) of GISTs have KIT mutations that are in-frame deletions or insertions, missense mutations, or combinations of both. The PDGFR mutation is present in about a third of wild-type KIT tumors [19]. These two mutations are mutually exclusive [133].

The tyrosine kinase inhibitors (TKIs) target almost 90% of the mutant form of both KIT and PDGFR. These drugs are very efficient, with a >80% objective response rate in patients [134], but most of them eventually develop acquired resistance [20]. Therefore, research has focused on alternative oncogenic pathways activated in these tumors. The PI3K/Akt pathway is part of the downstream factors of the tyrosine kinases (TKs) and has an important role in both predicting and promoting resistance to the inhibitors of the receptor TKs (RTKs) [135].

In vitro studies have established that imatinib-resistant GIST cell lines display strong KIT-dependent MAPK and Akt activation, with a central role of PI3K/Akt/mTOR in survival [136].

A mono-allelic PTEN loss occurs in 24% of GISTs, whereas bi-allelic PTEN loss is very uncommon [41]. Additionally, the reduction and/or absence of PTEN expression is detected in up to 50% of imatinib-treated GISTs. The reduction of PTEN expression is mainly associated with high-risk/metastatic tumors [41] and has a strong negative impact on overall survival [42]. Studies have confirmed that the combination of imatinib mesylate and PI3K inhibitors in the treatment of GIST xenograft models was more effective, with a more durable response compared to the use of a single-agent [134,137,138].

## 8. Epithelioid Sarcoma

Epithelioid sarcoma (ES) is a rare neoplasm accounting for <1% of adult STSs [139]. It has the peculiar characteristic of expressing both mesenchymal and epithelial markers, but although the cell origin is still debated, it is acknowledged to be a mesenchymal origin [140]. From the molecular point of view, this tumor has been shown to have a homozygous SMARCB1 (INI1) deletion in >90% of both the proximal and conventional subtypes, resulting in the complete absence of INI1 protein expression [141]. In vitro studies have established that the Akt/mTOR pathway is constitutively hyperactivated in SMARCB1-deficient cells [142,143]. PTEN expression on tissue samples is lost in 40% of these tumors, causing mTOR pathway activation [46]. The transfection of cell lines with anti-mTOR-specific siRNAs had an essential impact on cell proliferation. However, the inhibition of mTOR with an mTOR inhibitor (everolimus) reduced the tumor growth in xenograft mice, but did not shrink the tumor size, probably due to the increased activity of Akt and ERK by this drug, confirming the c-MET-dependent resistance of the tumor [142].

In vitro and in vivo studies have given some promising results, with the combined use of compounds targeting both Akt and c-MET activity [142] or the combination of mTOR and EGFR inhibitor [46], confirming the necessity of a multimodal treatment approach for these types of tumors.

## 9. Conclusions

Despite the advent of a more systemic approach in treatment strategies, due to their heterogeneity and rarity, STSs remain a major challenge for the clinicians. The conventional cytotoxic therapies improve survival in some subtypes of sarcomas, but in most of the high-grade advanced tumors, they have no or a minimal benefit [144]. In the past, STSs were treated as a unique disease, but pre-clinical and clinical research has shown that “lumping” sarcomas together does not make any sense, neither from a biological nor clinical perspective. In the age of personalized medicine, where cancers are treated as a unique disease in each patient, broad recognition of the tumor biology is essential and the identification of potential biomarkers for STS is gaining interest as the only way to improve life expectancy in the case of advanced tumors.

Recent translational studies and clinical trials have focused on a precise stratification of these tumors based on distinct histological features and solid molecular data. Therefore, it is becoming more common in specialized centers to include a further in-depth genetic analysis in the standard pathological assessment, including karyotyping and cDNA/protein expression profiling. The information provided can improve the ability to predict the biological behavior of the STS, and establish amenable molecular targets.

The research on a single gene(s) driving mutation sarcomas has focused on therapies with drugs that specifically inhibit the activated kinase receptor. The sarcomas with distinct translocations are more challenging since most of the fusion oncogenes encode transcription factors that cannot be targeted directly. Therefore, research has focused on the clinical targetable secondary alterations induced by the fusion proteins. The identification of feasible target genes for treatment in patients with STS harboring non-distinctive aberrant molecular alterations is very difficult. Their underlying genetic mechanisms involve multiple pathways and include alterations in several cell-cycle genes. The therapeutic interventions mainly focus on the effort to suppress the more commonly activated targetable pathways. It is reasonable to think that further evolution of the classification of these tumors based on a deeper understanding of the biological properties through detailed gene expression and proteomic analysis will improve our ability to predict future clinical behavior and allow more specific therapeutic interventions.

The PTEN/PI3K/mTOR pathway is one of the main focuses in cancer discovery because of the frequent dysregulation through the increased expression and/or activation of receptor tyrosine kinases, mutations in pathway components, or loss of negative pathway regulators, such as PTEN [145]. The members of this pathway have been intensely scrutinized as potential therapeutic targets and prognostic biomarkers in multiple types of STSs, with some promising results.

Sarcoma therapy will likely involve agents that target PTEN/ALK/MTOR pathway members alone, in combination, or/and with other oncogenic pathways associated with or without standard cytoreductive chemotherapy. For example, pazopanib, an FDA approved oral agent in high-grade STS, is a multikinase tyrosine kinase inhibitor that targets multiple receptors, such as the vascular endothelial growth factor (VEGF), platelet-derived growth factor (PDGFR), and c-KIT. It has been used in combination with mTOR or MEK inhibitors, with some interesting results [146].

PTEN remains one of the most important tumor suppressors, with an undisputed relevance in cancer, but due to complex regulation within the cell, its prognostic and predictive role remains very controversial. Inactivating mutations of *PTEN* take place in only a fraction of PTEN-deficient tumors. Additionally, PTEN function can be lost by several non-genomic mechanisms, such as epigenetic and transcriptional silencing, post-translational modifications (phosphorylation, acetylation, oxidation, and ubiquitylation), or by interaction with proteins that suppress its functions. Therefore, it is not surprising that several clinical trials have failed to demonstrate a correlation between PTEN loss and the clinical outcome of specific drugs, such as mTOR inhibitors [147,148,149,150,151,152]. A recent study suggested that the copy number loss or mutation of PTEN analyzed by DNA sequencing instead of IHC has a better predictive value to establish the patients which can gain some potential benefit from treatment [150,153].

Currently, no specific validated method is available for PTEN assessment; however, it can be clinically useful to determine its status in distinct STSs both by DNA sequencing and protein quantification by IHC, western-blot, or/and rt-PCR, as neither method alone provide complete information. Moreover, some of the downstream factors, such as Akt and the phosphatase activity of PTEN, should also be evaluated. The new-generation arrays help to analyze a large number of parameters, and in the future, they will be accessible in routine clinical assessment.

Finally, the exceptionally complex regulation of PTEN has stymied the progress for establishing agents that therapeutically enhance its tumor suppressive functions. Therefore, it is fundamental to conduct further comprehensive assessment of its status in different tumor subtypes, either when PTEN is lost or not lost. The sarcomas, with their genetically simple or/and complex subtypes and variety of oncogenic mechanisms that are involved, can provide a striking model to increase our understanding and encourage the progress in research and drug development [153].

## Figures and Tables

**Table 1:** Incidence of PTEN alterations in STTs.

Paper	Subtype of STTs	Molecular Mechanism(s) Of PTEN Alteration
Movva et al. [15]	38.6% of all STTs	PTEN loss of expression
	32.2% of non uLMSs	
	37.6% of uLMSs	
	29–44% UPSs, MPNSTs, LMSs	
Cote et al. [29]	10% of all STTs	PTEN gene mutation/deletion
	21% of LMSs	
	33% MLPSs	
	11% GISTs	
	7% UPSs	
	7% Chondrosarcomas	
	4% Chordomas	
Hernando et al. [30]	75% of LMSs	pAKT overexpression
	90% of MFH/UPSs	
	83% of DDLPSs	
Tomita et al. [31]	30% of LPSs	pAKT overexpression
	32% of MFHs	
	20% of LMSs	
	28% of MPNSTs	
	3% of UPSs	
Hu et al. [34]	58% of LMSs	10q deletion
Gibault et al. [35]	46% of STTs (LMS, MFS, PLPS, UPS)	PTEN partial/pronounced loss
	68% of STTs (LMS, MFS, PLPS, UPS)	Loss of expression (WB)
	26% of STTs	Loss of expression (IHC)
Cuppens et al. [36]	34% of all uterine sarcomas	PTEN loss of expression
	28% of LMSs	
Gutierrez et al. [37]	32% of WDLPSs	pAKT overexpression
	45% of DDLPSs	
	41% of WDLPSs	pS6 overexpression
	47% of DDLPSs	
Demicco et al. [38]	15% of MLPSs	PTEN loss of expression
	13% of MLPSs	Mutation of PIK3CA
Bradtmoller et al. [39]	42% of MPNSTs	PTEN promotor methylation
	MPNSTs compared to neurofibromas	PTEN loss of expression
Gregorain et al. [40]	78% of MPNSTs	PTEN loss of expression
Quattrone et al. [41]	24% of GISTs	Mono-allelic PTEN loss
	32% of GISTs	PTEN loss of expression
Ricci et al. [42]	42.8% of GISTs	PTEN loss of expression
Gibault et al. [43]	STSs with complex genomics	PTEN loss of expression
	50% of Group D* (MFS, UPS, PLPS)	Partial 10 q loss
Saito et al. [44]	14.3% of SSs	PTEN mutation
Teng et all. [45]	6.7% of SSs	PTEN mutation
	46.7% of SSs	PTEN loss of expression
Xie et al. [46]	80% of ESs	PTEN loss of expression

## Abbreviations

GIST	Gastrointestinal Stroma Tumor
MPNST	Malignant Peripheral Nerve Sheath Tumors
MLPS	Myxoid Liposarcomas
LMS	Leiomyosarcoma
MFS	Myxofibrosarcoma
WD/DDLPS	Well/Dedifferentiated Liposarcoma
RMA	Rhabdomyosarcoma
UPS	Unclassified Pleomorphic Sarcoma
